# Workforce migration and brain drain – A nationwide cross-sectional survey of early career psychiatrists in Nigeria

**DOI:** 10.1017/gmh.2024.25

**Published:** 2024-02-29

**Authors:** Emmanuel Aniekan Essien, Mohammed Yusuf Mahmood, Frances Adiukwu, Yesiru Adeyemi Kareem, Nafisatu Hayatudeen, Margaret Isioma Ojeahere, Mumeen Olaitan Salihu, Kamaldeen Adeyinka Sanni, Ayotunde Bolatito Omotoso, Mariana Pinto da Costa

**Affiliations:** 1 Department of Clinical Services, Federal Neuropsychiatric Hospital, Calabar, Nigeria; 2 Department of Clinical Services, Federal Neuropsychiatric Hospital, Maiduguri, Nigeria; 3 Department of Mental Health, University of Port Harcourt, Choba, Rivers State, Nigeria; 4 Directorate of Clinical Services, Neuropsychiatric Hospital, Aro, Abeokuta, Nigeria; 5 Department of Clinical Services, Federal Neuropsychiatric Hospital, Kaduna, Nigeria; 6 Department of Psychiatry, Jos University Teaching Hospital, Plateau, Nigeria; 7 Department of Behavioural Sciences, University of Ilorin Teaching Hospital, Ilorin, Nigeria; 8 Institute of Psychiatry, Psychology & Neuroscience, King’s College London, London, UK

**Keywords:** brain drain, human migration, health personnel, psychiatry, Nigeria, workforce migration

## Abstract

**Background:**

Nigeria’s shortage of psychiatrists is exacerbated due to health worker migration.

**Aim:**

This study explores migration experiences and tendencies among early-career psychiatrists in Nigeria.

**Methods:**

We conducted a cross-sectional survey covering Nigeria’s six geopolitical zones, using a 61-item online questionnaire assessing short-term mobility, long-term migration experiences and migration attitudes. Data was analysed using IBM SPSS version 29.

**Results:**

Of 228 early-career psychiatrists surveyed, 9.7% had short-term mobility and 8.0% had long-term migration experiences. However, 85.8% had ‘ever’ considered migration, 69.2% were planning to leave ‘now’, and 52.9% had taken ‘practical migration steps’. Over half (52.7%) said they would be working abroad in 5 years, with 25.2% indicating they would migrate within a year. The top reasons to leave were financial and academic, while personal and cultural factors were the key reasons to stay. Income dissatisfaction (OR = 2.27, 95%, CI = 1.05–4.88) predicted planning to leave ‘now’, while being in a relationship (OR = 3.46, 95%CI = 1.06–11.30) predicted taking ‘practical migration steps’. Attractive job features were good welfare (85.4%) and high salaries (80.3%). Improvements in finances (90.8%) and work conditions (86.8%) were requested.

**Conclusions:**

Systemic changes to address psychiatrists’ migration from Nigeria are needed.

## Impact statement

Mental health care is deprioritised in Nigeria compared to other medical specialities, and over 90% of people with psychiatric disorders are either undiagnosed or untreated, despite the availability of effective and affordable treatments. Importantly, the number of psychiatrists in the country is grossly inadequate, with around 250 caring for a population of over 200 million. In the aftermath of the COVID-19 pandemic, there was an upsurge in health worker migration from Nigeria, further depleting the number of psychiatrists in the country. However, to date, little is known about migration tendencies and their motivating factors among psychiatrists in Nigeria. We aimed to fill this gap by conducting a nationally representative survey covering over 70% of early career psychiatrists from Nigeria’s six geopolitical regions. We found that over half had taken practical steps towards migration and predicted they would be working outside the country in 5 years. Losing such a high number of psychiatrists might prove disastrous for the country’s fragile mental health care service. Our results underscore the urgent need for the Nigerian government to implement system-wide improvements to curb health worker migration and increase the retention of psychiatrists in Nigeria.

## Introduction

In 2020, approximately 281 million people, corresponding to 3.60% of the global population, lived outside their country of birth – a marked increase from an estimated 128 million in 1970 (McAuliffe and Triandafyllidou, 2022). Many of these migrants are highly skilled professionals transferring their expertise from ‘donor’ to ‘recipient’ countries, a phenomenon known as ‘brain drain’ (Pinto da Costa et al., 2017). Health worker migration is a longstanding trend with positive outcomes for medical services in destination countries while adversely affecting the health systems of donor nations (World Health Organization, 2023a).

Various “push” and “pull” factors drive health worker migration (Al-Khalisi, 2013). “Push” factors arise from the home country and include poor work conditions, limited opportunities for career development, unsatisfactory wages, poor job satisfaction, economic hardship, sociopolitical unrest, insecurity, concern for children’s future and poor living standards (Ogaboh et al., 2020; Toyin-Thomas et al., 2023). Conversely, “pull” factors emanate from the destination countries and comprise better wages, improved career opportunities, a good work environment, a better quality of life, security and active recruitment (Toyin-Thomas et al., 2023). Both factor categories exert their influence on three primary levels: micro-level (personal factors), macro-level (national and cross-national factors) and meso-level (related to one’s profession) (Toyin-Thomas et al., 2023).

The COVID-19 pandemic overwhelmed health systems worldwide, revealing pre-existing care and staffing deficiencies, causing a surge in health worker migration (Poon et al., 2022). In the global south, 55 countries now rank below the global median number of doctors and nurses *per capita*, increasing by eight countries compared to 2020 data (World Health Organization, 2023a). Nations with the lowest health worker densities also tend to have the highest burden of disease, measured in disability-adjusted life years (World Health Organization, 2023b). Brain drain increases the vulnerability of weak health systems, where health workers are critically needed to provide essential services (World Health Organization, 2023a). The impact of health worker migration on psychiatric services in the global south might be worse compared to other medical specialities, given the stigmatisation and under-prioritisation of mental health conditions in developing countries (Semrau et al., 2015). Although effective and safe treatments are available, the mental disorder treatment gap reaches 90% in Sub-Saharan Africa (Mugisha et al., 2017).

Nigeria, the most populous country in the region, has about 250 certified psychiatrists serving over 200 million people, compared to 4,500 full-time psychiatrists serving 56.5 million people in the United Kingdom (Okechukwu, 2020; Royal College of Psychiatrists, 2021). Even though Nigeria has been training its own psychiatrists since the 1960s, the number available in the country remains less than one psychiatrist per million due to brain drain (Bakare and Bakare, n.d.; Jibunoh and Ani, 2022). In 2010, there were 384 Nigeria-trained psychiatrists in the United States, United Kingdom, New Zealand and Australia alone, a number that was (and still is) higher than those practising in Nigeria (Jenkins et al., 2010). In an interview, a medical director of one of the foremost psychiatric hospitals in Nigeria asserted that the brain drain of psychiatrists in the country has markedly increased post-pandemic, with a loss of around 100 Nigerian psychiatrists in the last 3 years (Fatunmole, 2022). Further worsening the disadvantage, psychiatry is one of the least preferred choices for post-graduate specialisation among graduating medical students (Ossai et al., 2016).

Several sub-Saharan African countries face challenges due to a shortage of psychiatrists. In East Africa, Kenya has only 115 psychiatrists serving a population of over 50 million (Marangu et al., 2021). In West Africa, Ghana, with a population of 28 million people, has only 14 psychiatrists, while South Africa, in the southern part of the continent has 850 psychiatrists attending to a population of over 55 million (Agyapong et al., 2020; Rensburg et al., 2021). The overall situation in Sub-Saharan Africa is worse compared to developed nations, despite variations among individual countries. According to WHO data, 32 out of 39 countries in the region have fewer than 0.5 psychiatrists per 100,000. In contrast, only 4 out of 28 in the Americas and none out of 39 in Europe have a similar low number of psychiatrists (Rensburg et al., 2021).

There have been attempts at the international and local levels to mitigate the impact of health worker migration. The World Health Organisation developed a global code of practice in 2010 for the international recruitment of health personnel (Hinlopen et al., 2020; Toyin-Thomas et al., 2023). The aim was to provide an ethical framework for health worker recruitment and strengthen national health workforces to reduce the reliance of richer countries on health workers from poorer nations; however, implementation has been poor (Hinlopen et al., 2020; Toyin-Thomas et al., 2023). In Nigeria, there was a recent controversial attempt by the National Assembly to restrict the migration of doctors after graduation (Adejoro, 2023). However, the ‘Anti-migration bill’ faced stiff opposition from medical professionals and has yet to be passed (Adejoro, 2023).

This article aims to comprehensively assess migration experiences, tendencies and driving factors in early career psychiatrists in Nigeria.

## Methods

### Study design

Cross-sectional survey.

### Eligibility criteria

Early career psychiatrists in Nigeria were eligible for inclusion and were defined as: i) psychiatry trainees enrolled in a nationally accredited psychiatry training program in Nigeria and ii) psychiatrists within 7 years of completing their speciality training in Nigeria.

### Study instrument

A 61-item questionnaire was used, inquiring about:

- Socio-demographic characteristics
- Short-term mobility (between 3 months and 1 year).
- Long-term migratory experiences (lasting over 1 year).
- Migration tendencies.

More details about the questionnaire have been published elsewhere (Pinto da Costa et al., 2017, 2019).

### Data collection

Data were collected between December 2022 and November 2023 across the six geopolitical zones in Nigeria (the North-east, North-west, North-central, South-south, South-east and South-west). There were 192 psychiatric trainees and 119 (early career) psychiatrists (ECPs) in Nigeria at the time of the study according to the official register of the Association of Psychiatrists in Nigeria - Early Career Psychiatrists Section (APN-ECPs). The questionnaire was distributed online using social networks of early career psychiatrists in Nigeria (Survey Monkey v. 15 November 2022 http://www.surveymonkey.net). The study was anonymous, and only participants who met the inclusion criteria and gave informed consent were included in the study.

### Data analysis

Data were analysed using IBM SPSS Statistics for Windows, Version 29.0. Categorical variables, such as socio-demographic characteristics, were reported as frequencies and proportions, whereas continuous variables were summarised as means. Missing data were omitted in the complete case analysis, and only valid percentages were reported. Three categorical variables with an increasing hierarchy of ‘migratory tendency’ were assessed as follows: (i) ‘ever’ considered leaving the country (Yes/No); (b) Considering leaving the country ‘now’ (responses of ‘strongly agree’ or ‘agree’ were coded ‘Yes’, others coded ‘No’; (c) taking ‘practical steps’ to leave the country (Yes/No).

In multivariate analysis, generalised estimating equations accounted for clustering by geopolitical zones in Nigeria. Socio-demographic characteristics were assessed as predictors of each level of migratory tendency. Relationship status (single, married, in a relationship, divorced) was re-coded as a dichotomous variable ("in a relationship" and “not in a relationship”. Living arrangements (living alone, with family, with colleagues, with others) were also dichotomised ("living alone" and “living with others”). The alpha threshold for statistical analysis was 0.05.

## Results

### Socio-demographic characteristics

A total of 228 respondents out of 311 participated in the study, yielding a response rate of 73.3% (72.9% for psychiatric trainees and 73.9% for ECPs). Most were male (n = 125, 60.7%), and the sample mean age was 35.86 (SD = 5.50). The majority of participants were in a relationship (n = 177, 85.9%), had children (n = 152, 73.8%) and lived with their families (n = 161, 80.1%). Most (n = 130, 64.7%) lived in rented apartments, and some owned their accommodation (n = 27, 13.4%). Almost two-thirds were still in their training (n = 140, 61.4%). For the vast majority (n = 182, 90.5%), the monthly income was less than a million naira (i.e., less than €999). The least populated monthly income categories were those who earned between 1 million and 1.5 million naira, that is, €1,000–€1,499 (n = 8, 4.0%), followed by those who earned between 1.5 million and 3 million naira, that is, €1,500–3,000 (n = 11, 5.5%). Furthermore, about a fifth (n = 39, 19.4%) had additional income. The majority (n = 133; 66.2%) were either dissatisfied or very dissatisfied with their income, while less than a fifth (n = 28, 13.9%) were either satisfied or very satisfied. More information about the sample characteristics is displayed in Table 1.

**Table 1.** Socio-demographic data of respondents

Variable	*N* (%)
Gender	
Male	125 (60.7)
Female	81 (39.3)
Age	35.86 (5.50); range 26–53
Relationship status	
Married	163 (79.1)
In a relationship	14 (6.8)
Single	28 (13.6)
Divorced	1 (0.4)
Children	
Yes	152 (73.8)
No	54 (26.2)
Living arrangements	
Alone	33 (16.4)
With family	161 (80.1)
With friends	2 (1.0)
With colleagues	3 (1.5)
Homeownership status	
Owner of your house	27 (13.4)
Parent’s house	16 (8.0)
Rented	130 (64.7)
Free accommodation	6 (3.0)
Hospital accommodation	22 (10.9)
Professional role	
Psychiatry trainee	140 (61.4)
Early career psychiatrist	88 (38.6)
Income (in Euros)	
<250	48 (23.9)
250–499	71 (35.3)
500–999	63 (31.3)
1,000–1,499	8 (4.0)
1,500–3,000	11 (5.5)
Additional income (in Euros)	
Yes	39 (19.4)
No	162 (80.6)
Income satisfaction	
Very satisfied	3 (1.5)
Satisfied	25 (12.4)
Neither satisfied nor dissatisfied	40 (19.9)
Dissatisfied	78 (38.8)
Very dissatisfied	55 (24.1)

### Short-term mobility

Only a few respondents (n = 22, 9.7%) had previous short-term mobility experiences abroad, with the number of lifetime experiences ranging from 1 (n = 10) to 10 (n = 1). Most travelled for education (n = 10), followed by work-related travel (n = 5), and none travelled for volunteering. The most visited countries for the first short-mobility experience were the UK (n = 4) and Germany (n = 2) and most (n = 17, 77.2%) of those who travelled were either satisfied or very satisfied with their first short-mobility experience. A similar trend was present among the few (n = 7) with a second short-mobility experience. Among those who had any short mobility experience, for some (n = 14), these experiences impacted their attitude towards migration, while there was no impact in seven. The effect on migration attitudes was negative in four participants and positive in 10.

### Long-term migration experience

Eighteen respondents (8.0%) reported long-term migration once in their lifetime. Most migrated during their university education (n = 7), while others travelled during their psychiatric training (n = 5) or after their psychiatric training (n = 4). The United Kingdom was the country most visited during these experiences (n = 7), followed by Russia (n = 2). The top reasons for migration were academic (n = 9), financial (n = 3), personal (n = 4), political (n = 2), religious (n = 1), cultural (n = 3), social (n = 3) and work (n = 2). The mean duration of long-term migration was 62.93 (SD = 24.66) months. Half migrated alone (n = 8), while the remainder migrated with family (n = 8). Most (n = 14) were satisfied or very satisfied with the experience, and none were dissatisfied. Six of the migrants felt they had equal opportunities as the locals, but some reported inequities in work (n = 4), financial (n = 4), social (n = 2), cultural (n = 4), political (n = 4), religious (n = 3) and personal (n = 2) aspects.

### Migration tendency and future work prospects

Over three-quarters of respondents (n = 187, 85.8%) had ‘ever’ considered leaving Nigeria, more than two-thirds (n = 128, 69.2%) were considering leaving ‘now’, while over half (n = 92, 52.9%) had taken ‘practical steps’ towards migration. Nearly a third (n = 54, 29.2%) started thinking about migration in the previous 6 months, another third (n = 54, 29.2%) had been considering migration for over 6 months but less than 1 year, and the remainder (n = 77, 41.6%) had been thinking about it for over a year.

Across the sample, about half (n = 115, 52.7%) considered that they would be working outside Nigeria in 5 years’ time, either in Europe (n = 41, 18.8%) or elsewhere in the world (n = 74, 33.9%). Nearly a third (n = 52, 29.5%) said they would likely be in the country in 5 years, whereas a fifth were undecided (n = 36, 20.5%).

Among those planning to migrate, some (n = 29, 25.2%) were planning to leave in a year, while the majority (n = 86, 74.8%) were yet to make arrangements. Sources most used for job search were personal networks (n = 52, 22.8%), job search engines (n = 42, 18.4%), professional networks (n = 43, 18.9%), employment agencies (n = 37, 16.2%) and general online search (n = 24, 10.5%).

Over two-thirds strongly agreed that features of an attractive job were good welfare and social security (n = 182, 85.4%), followed by high salary (n = 171,80.3%), pleasant working environment (n = 166, 77.9%), opportunities for professional growth (n = 165, 77.5%), work–life balance (n = 166, 77.9%), the ability to contribute to the community (n = 145, 68.1%) and acknowledgement for their efforts (n = 131, 61.5%). ‘Less workload’ (n = 83, 39.0%) and ‘work independence’ (n = 29, 13.6%) were the least ranked reasons for job attractiveness (see Figure 3).

### Reasons to leave or stay in the country

**Figure 1.**
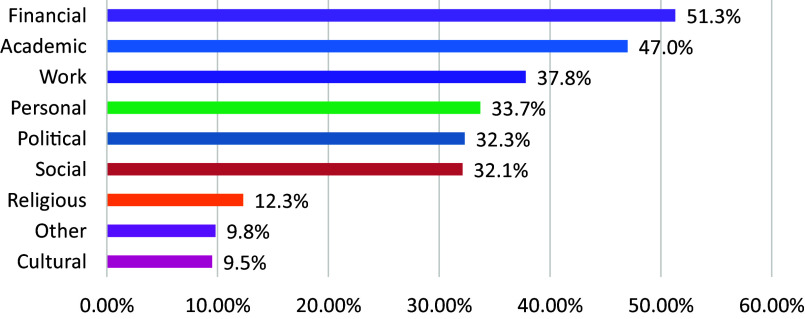
Reasons to leave.

**Figure 2.**
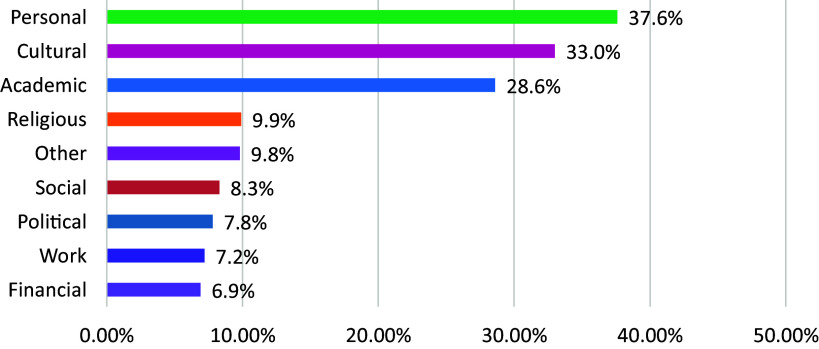
Reasons to stay.

**Figure 3.**
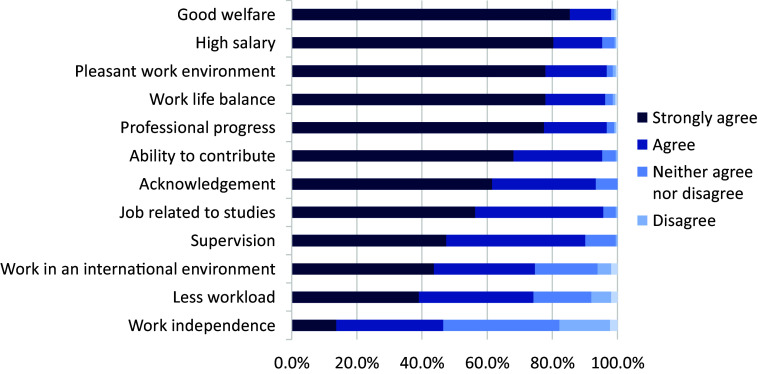
Features of an attractive job.

The main reasons to leave the country were financial (n = 102, 51.3%), academic (n = 93, 47.0%), personal (n = 66, 33.7%) and political (n = 62, 32.3%), while the main reasons for staying were personal *e.g.* relating to health, partner, children, family (n = 74, 37.6%), cultural (n = 65, 33.0%), academic (n = 57, 28.6%) and social (n = 19, 9.9%). Further details are shown in Figures 1, 2 and 3. Participants reported that improvements in local psychiatry were needed in finances (n = 207, 90.8%), work conditions (n = 198, 86.8%), academics (n = 170, 74.6%) and professional networking (n = 143, 62.7%).

### Migration tendency across the geopolitical zones

A higher proportion of respondents had ‘ever’ considered migration in the North-central region (n = 25, 96.2%), compared to the South-west (n = 40, 93.0%), South-east (n = 12, 92.3%), North-east (n = 22, 84.5%), North-west (n = 37, 80.4%) and South-south (n = 49, 79.0%). In the North-west, there were more psychiatrists considering leaving the country ‘now’ (n = 15, 86.1%), compared to the South-west (n = 30, 76.9%), South-east (n = 9; 75%), North-east (n = 15, 68.2%), South-south (n = 28, n = 57.1%) and North-central (n = 14, 56.0%). Furthermore, a higher proportion had taken ‘practical steps’ in the South-west (n = 28, 73.7%), followed by the South-east (n = 6, 54.5%), North-west (n = 18, 51.4%), North-east (n = 9, 50.0%), South-south (n = 22, 45.8%) and North-central (n = 8, 36.4%).

### Predictors of migratory tendencies

Table 2 reports a multivariable analysis of individual characteristics (income satisfaction, age, gender, income level, professional stage, having children, relationship status and living arrangements) as predictors of migration tendencies. In the first multivariable model assessing predictors of ‘ever’ considering leaving, none of the characteristics emerged as a significant predictor. However, in the second model assessing predictors of considering leaving the country ‘now’, only income dissatisfaction emerged as a predictor (p < 0.05). In the third model, only being in a relationship emerged as a predictor of taking practical steps towards migration (p < 0.05).

**Table 2.** Multivariable analysis of individual characteristics associated with migration tendency

Migration tendency	B	SE	WALD	*p*	OR	95% CI	
						Lower	Upper
Have you ‘ever’ considered leaving the country you live in?							
Income satisfaction^a^	0.81	0.43	3.42	0.06	2.24	0.95	5.30
Age	−0.05	0.03	1.55	0.21	0.95	0.88	1.02
Gender^b^	0.04	0.42	0.01	0.91	1.04	0.45	2.43
Income^c^	−0.06	0.51	0.01	0.89	0.93	0.34	2.54
Profession stage^d^	−0.34	0.44	0.58	0.44	0.71	0.29	1.69
Children^e^	−0.47	1.05	0.20	0.65	0.62	0.07	4.86
Relationship status^f^	−0.01	1.25	0.00	0.98	0.98	0.08	11.58
Living arrangement^g^	−0.19	0.70	0.07	0.78	0.82	0.20	3.27
I am considering leaving the country ‘now’							
Income satisfaction^a^	0.82	0.39	4.43	**0.03**	2.27	1.05	4.88
Age	0.02	0.04	0.30	0.58	1.02	0.94	1.11
Gender^b^	0.63	0.36	2.91	0.08	1.87	0.91	3.87
Income^c^	0.48	0.42	1.29	0.25	1.62	0.70	3.73
Profession stage^d^	0.61	0.37	2.71	0.10	1.85	0.88	3.86
Children^e^	0.72	0.62	1.33	0.24	2.05	0.60	6.96
Relationship status^f^	−1.23	0.70	3.01	0.08	0.29	0.07	1.17
Living arrangement^g^	−0.75	0.61	1.50	0.22	0.47	0.14	1.56
Did you take any ‘practical steps’ towards migration?							
Income satisfaction^a^	0.57	0.37	2.28	0.13	1.77	0.84	3.72
Age	−0.01	0.03	0.19	0.66	0.98	0.91	1.05
Gender^b^	−0.20	0.33	0.38	0.53	0.81	0.42	1.56
Income^c^	−0.69	0.38	3.32	0.06	0.50	0.23	1.05
Profession stage^d^	0.24	0.35	0.49	0.48	1.27	0.64	2.54
Children^e^	−0.24	0.57	0.18	0.66	0.78	0.25	2.41
Relationship status^f^	1.24	0.60	4.25	**0.03**	3.46	1.06	11.30
Living arrangement^g^	−0.43	0.47	0.84	0.35	0.64	0.25	1.64

B, coefficient; CI, confidence interval; OR, odds ratio; *p*, *p*-value; SE, standard error; WALD, Wald Chi-Square.

Bold *p*-values (α = 0.05) indicate statistical significance.

a dissatisfied with income.

b male gender.

c income less than £500.

d psychiatric trainee.

e have children.

f in a relationship.

g living with family.

## Discussion

### Key findings

The majority of respondents leaned towards migration out of Nigeria. Over three-quarters had ‘ever’ considered leaving Nigeria, two-thirds were considering leaving ‘now’, and half had taken ‘practical steps’ towards migration and predicted they would be out of the country in 5 years’ time. Job dissatisfaction was a predictor of thinking about leaving ‘now’, while being in a relationship predicted taking ‘practical steps’ towards migration. The main features for job attractiveness were social security, a pleasant work environment, and a good salary.

About two-thirds of respondents were either dissatisfied or very dissatisfied with their income. The top two reasons for leaving were financial and academic, while the main reasons for wanting to stay were personal and cultural. The key recommendations for local practice were improvements in financial conditions, work conditions, academics and professional networks.

Less than a tenth of the early career psychiatrists surveyed had previous short-term mobility experiences, primarily for education and work, and in over half, these experiences made them more inclined towards migration. Even fewer had long-term migration experiences, primarily for academic or personal reasons.

### Strengths and limitations

This is the first nationally representative study assessing the migration attitudes of early career psychiatrists in Nigeria, capturing over 70% of the study population. However, this study has some limitations. First, since the questionnaires were filled out voluntarily, there might be some self-selection bias with a possible overrepresentation of those interested in the subject of migration. Second, there might be social desirability bias, with respondents providing responses they consider more socially appropriate. The use of a self-report questionnaire could have also caused recall bias.

### Comparison with the literature

In our study, the majority (85.8%) of early career psychiatrists in Nigeria had ‘ever’ considered migration, a rate similar to findings in Ireland (90.3%) (Azvee et al., 2021), the Baltics (87.0%) (Matutyte et al., 2021), Italy (84.2%) (Orlando et al., 2022), Iran (83.7%) (Eissazade et al., 2021), Spain (77.7%) (Molina-Ruiz et al., 2022), Turkey (75.0%) (Kilic et al., 2019), Portugal (75.0%) (Pinto da Costa et al., 2021) and a European average of 72.0% (Pinto da Costa et al., 2017). Furthermore, in our study, 69.2% had considered leaving ‘now’, which was less than the rate in Ireland (76.3%) (Azvee et al., 2021), but higher than in Italy (60.4%) (Orlando et al., 2022), Iran (57.3%) (Eissazade et al., 2021), Turkey (55.6%) (Kilic et al., 2019), Spain (51.0%) (Molina-Ruiz et al., 2022), the Baltic countries (55.0%) (Matutyte et al., 2021), Portugal (49.0%) (Pinto da Costa et al., 2021) and the European average of 53.5% (Pinto da Costa et al., 2017). Importantly, more respondents in Nigeria had taken ‘practical steps’ towards migration (52.9%) than any other country previously investigated, such as 47.7% in Ireland (Azvee et al., 2021), 30.0% in Portugal (Pinto da Costa et al., 2021), 29.5% in Spain (Molina-Ruiz et al., 2022), 27.7% in Iran (Eissazade et al., 2021), 25.3% in Italy (Orlando et al., 2022), 12.3% in the Baltic countries (Matutyte et al., 2021), 7.6% in Turkey (Kilic et al., 2019) and a European average of 28.6% (Pinto da Costa et al., 2017). Similarly, more in our sample (52.7%) predicted that they would be outside the country in 5 years compared to 45% in Spain (Molina-Ruiz et al., 2022), 41.6% in Italy (Orlando et al., 2022), 34.6% in Ireland (Azvee et al., 2021), 26.8% in Portugal (Pinto da Costa et al., 2021) and 16.7% in Iran (Eissazade et al., 2021). While ‘ever’ considering migration was similar to other compared countries, planning to leave ‘now’ was second only to Ireland, a high-income country whose trainees are among the highest paid in Europe (Azvee et al., 2021). This is probably because Ireland is said to have a ‘culture of medical migration’ where international medical experience is prioritised by potential employers (Humphries et al., 2017). More Nigerians had, however, taken practical steps and predicted they would be outside the country in 5 years compared to the other countries. This is unsurprising as Nigeria has more developmental challenges and would likely have poorer work conditions and living standards (Wilson et al., 2013).

Financial considerations were the top-ranking push factor in our sample, similar to Spain (Molina-Ruiz et al., 2022), Portugal (Pinto da Costa et al., 2021) and the average in a multi-country European study (Pinto da Costa et al., 2017). In contrast, the main push factors were academic reasons in Italy (Orlando et al., 2022), Ireland (Azvee et al., 2021) and Turkey (Kilic et al., 2019) and politics in Iran (Eissazade et al., 2021). Given Nigeria’s weaker economy and poorer living standards, it is unsurprising that financial considerations would be the primary push factor. The most important pull factor in Nigeria was personal reasons, similar to Italy (Orlando et al., 2022), Iran (Eissazade et al., 2021), Spain (Molina-Ruiz et al., 2022) and Portugal (Pinto da Costa et al., 2021). The consistency of this factor across contexts demonstrates the importance of individual idiosyncrasies, for example, relating to health, partner, children and family, as influencers of migration. Good welfare and social security were the main features of an attractive job, similar to Iran (Eissazade et al., 2021), a middle-income country like Nigeria, but contrasting high-income countries like Italy (Orlando et al., 2022), Portugal (Pinto da Costa et al., 2021), the Baltics (Matutyte et al., 2021) and Ireland (Azvee et al., 2021), where a pleasant work environment was most important.

In Nigeria, fewer early career psychiatrists had short-term mobility experiences (9.7%) compared to 13.1% in Turkey (Kilic et al., 2019), 13.5% in Iran (Eissazade et al., 2021), 17.4% in Italy (Orlando et al., 2022), 28.4% in the Baltics (Matutyte et al., 2021), 30.6% in Portugal (Pinto da Costa et al., 2021), 45.2% in Ireland (Azvee et al., 2021) and 50.5% in Spain (Molina-Ruiz et al., 2022). Furthermore, 8.0% had long-term migration experiences compared to 2.5% in Italy (Orlando et al., 2022), 8.3% in Portugal (Pinto da Costa et al., 2021), 13.7% in Iran (Eissazade et al., 2021) and 35.6% in Ireland (Azvee et al., 2021). Migration is expensive, and therefore, doctors in weaker economies like Nigeria might be less able to afford it, possibly explaining their fewer short- and longer-term mobility experiences.

Income was lowest in Nigeria, with 90.5% earning less than €999, higher than 78.1% and 44.9% earning less than €999 in the Baltics (Matutyte et al., 2021) and Iran (Eissazade et al., 2021), respectively. Also, early career psychiatrists in other countries earned higher, with over 50% earning €1,000–1,499 in Turkey (Kilic et al., 2019), 86.4% earning € 1,000–€1,499 in Portugal (Pinto da Costa et al., 2021), 89.7% earning between € 1,500–1,999 in Italy (Orlando et al., 2022), all in Spain earning 1,500–2000 €/month (Molina-Ruiz et al., 2022) and 82.2% making over €2000 in Ireland (Azvee et al., 2021). While 19.4% had additional income in our study, it was 11.2% in Italy (Orlando et al., 2022), 17.9% in Iran (Eissazade et al., 2021), 24.0% in Portugal (Pinto da Costa et al., 2021) and 52.7% in the Baltics (Matutyte et al., 2021).

In our Nigerian sample, the lowest represented earning category – who were also high earners – were those with incomes between 1,000 and 1,499 (5.5%). However, the lowest represented groups in the compared nations were € 2,000–2,499 (n = 2, 1.7%) in Italy (Orlando et al., 2022) who were also high earners; € 500–999 (n = 2, 1%) in Iran (Eissazade et al., 2021) earning mid-range salaries and € 1,500–1999 (n = 1, 1.1%) in the Baltic countries (Matutyte et al., 2021), who were also high earners. The highest earners are expected to have the smallest numbers, consistent with the pyramid-shaped income distribution seen in most economies (Lopes, 2005).

In Nigeria, 66.2% were either dissatisfied or very dissatisfied with their income, a lower proportion than in Iran (82.1%) (Eissazade et al., 2021) but similar to rates reported in Turkey (58.0%) (Kilic et al., 2019), Ireland (56.3%) (Azvee et al., 2021) and Portugal (55.7%) (Pinto da Costa et al., 2021). Lower rates were reported in the Baltics (44.0%) (Matutyte et al., 2021), Italy (19.8%) (Orlando et al., 2022) and Spain (13%) (Molina-Ruiz et al., 2022). The most crucial area needing improvement in Nigeria was financial, similar to Iran (Eissazade et al., 2021), Spain (Molina-Ruiz et al., 2022) and the Baltics (Matutyte et al., 2021), whereas it was academics in Italy (Orlando et al., 2022) and Turkey (Kilic et al., 2019) and working conditions in Portugal (Pinto da Costa et al., 2021). Nigeria’s broad similarities to Iran could stem from both being weaker economies relative to the other compared countries.

The Nigerian sample tended to be older, with a mean age of 35.86 years (compared to 33.8 in Ireland (Azvee et al., 2021), 34.9 in Iran (Eissazade et al., 2021), 30.09 in Italy (Orlando et al., 2022), 29.0 in Spain (Molina-Ruiz et al., 2022), 28.7 in Portugal (Pinto da Costa et al., 2021), 29.43 in the Baltics (Matutyte et al., 2021) and 28.0 in Turkey (Kilic et al., 2019) and had less women (39.3%) compared to other countries, such as the Baltics (79.1%) (Matutyte et al., 2021), Iran (73.4%) (Eissazade et al., 2021), Turkey (72.8%) (Kilic et al., 2019), Ireland (64.6%) (Azvee et al., 2021), Italy (64.1%) (Orlando et al., 2022), Portugal (60.6%) (Pinto da Costa et al., 2021) and Spain (58%) (Molina-Ruiz et al., 2022). The majority in Nigeria had children (73.8%) compared to only 5.7% in Portugal (Pinto da Costa et al., 2021), 8.6% in Turkey (Kilic et al., 2019), 14.5% in Italy (Orlando et al., 2022), 32.1% in Iran (Eissazade et al., 2021), 37.5% in Ireland (Azvee et al., 2021) and 38.5% in the Baltics (Matutyte et al., 2021). In Nigeria, 85.9% were in relationships (including those who were married) compared to 58.8% in Portugal (Pinto da Costa et al., 2021), 65.4% in Turkey (Kilic et al., 2019), 71.2% in Iran (Eissazade et al., 2021) and 77.1% in Ireland (Azvee et al., 2021).

Our finding in Nigeria that income dissatisfaction predicted wanting to leave the country ‘now’ aligns with findings from a prior study involving 33 European countries (Pinto da Costa et al., 2017). However, income dissatisfaction did not predict ‘ever’ considering or taking ‘practical steps’ towards migration. Despite income dissatisfaction, the low wages in Nigeria might hinder taking ‘practical steps’ towards migration because migration costs far outweigh the health worker’s earnings. Furthermore, in contrast with the European multi-country study, we found that being in a relationship predicted taking practical migration steps. The study from the Baltic countries instead found that being single significantly predicted taking practical migration steps (Matutyte et al., 2021). Unlike these studies, most in a relationship in our study in Nigeria were married and had children. These socio-demographic disparities might shape migration decisions, prioritising the welfare of the whole family, especially that of the children.

## Implication of the findings for practice, policies and research

The WHO lists Nigeria among the 55 nations facing critical health worker shortages and inadequate universal health coverage (World Health Organization, 2023a). If unaddressed, further migration of health workers might have dire implications for the already fragile Nigerian health care system. This study fills a crucial data gap concerning the migration tendencies of psychiatric trainees and early career psychiatrists in Nigeria, shedding light on the reasons driving their emigration and highlighting local aspects that should be improved. Our results are also relevant beyond the psychiatric speciality, providing insights into the current tendencies of the Nigerian health worker towards migration.

Financial concerns, including income dissatisfaction, were the most critical factors fostering migration propensity in this study. Most psychiatrists in Nigeria are public sector workers, and the finance ministry centrally determines salaries (Wada et al., 2021). A protracted struggle between national trade unions and the government aimed at securing increasing salaries, marked by frequent strikes, has been ongoing (Opone and Kelikwuma, 2021). However, improving the wages of health workers in Nigeria might be challenging to implement, as the nation is facing an economic crisis and struggles to pay salaries (Micah and Moses, 2018).

Academic reasons also ranked high among the reasons for migration in our study. A previous survey in Nigeria among junior doctors revealed dissatisfaction with the quality of their medical training, with the top reason for migration being the pursuit of their clinical education (Akinyemi et al., 2022). Improving the academic quality and standardising the post-graduate training curriculum could contribute to the retention of psychiatrists in Nigeria.

The ethical dilemma of recruiting health workers from regions with critical staff shortages, such as the global south, calls for a balance between migration as a fundamental human right and the population’s right to primary health care. Nonetheless, the government’s responsibility includes implementing statewide improvements in wages, training quality and social security to incentivise health workers’ retention, including psychiatrists, in Nigeria. However, considering the substantial investments developing nations like Nigeria make in training their health workers, an argument can be made for wealthier destination countries to compensate the poorer donor nations for each health worker they receive. These funds could then be used to improve the health systems of donor nations and facilitate the training of additional medical professionals.

## Conclusions

In Nigeria, early career psychiatrists have low short-term mobility and long-term migration experiences. However, there is a high migration tendency, with about half of the respondents already taking practical migration steps and planning to be outside the country in 5 years. Poor remuneration was the top reason driving migration, while the main reasons to stay in the country were personal. Our results suggest that Nigeria is on the verge of substantial psychiatrists’ emigration, posing significant challenges to the country’s mental health service.

## Data Availability

De-identified data for this manuscript may be made available upon reasonable request to the authors and the completion of an appropriate data transfer agreement.
