# Supplementary material for: Workforce migration and brain drain – A nationwide cross-sectional survey of early career psychiatrists in Nigeria
Source: Glob Ment Health (Camb). 2024 Feb 29;11:e30. doi: 10.1017/gmh.2024.25 (PMC10988168; doi:10.1017/gmh.2024.25)
Supplement: Peer review history

# Editor report: Recommendation: Workforce migration and brain drain – A nationwide cross-sectional survey of early career psychiatrists in Nigeria — R0/PR2

**DOI:** 10.1017/gmh.2024.25.pr2

**Affiliations:** Centre for Rural Health, University of KwaZulu-Natal, South Africa

No accompanying comment.

# Editor report: Decision: Workforce migration and brain drain – A nationwide cross-sectional survey of early career psychiatrists in Nigeria — R0/PR3

**DOI:** 10.1017/gmh.2024.25.pr3

**Affiliations:** London School of Hygiene & Tropical Medicine, United Kingdom of Great Britain and Northern Ireland

No accompanying comment.

# Editor report: Recommendation: Workforce migration and brain drain – A nationwide cross-sectional survey of early career psychiatrists in Nigeria — R1/PR5

**DOI:** 10.1017/gmh.2024.25.pr5

**Affiliations:** Centre for Rural Health, University of KwaZulu-Natal, South Africa

No accompanying comment.

# Editor report: Decision: Workforce migration and brain drain – A nationwide cross-sectional survey of early career psychiatrists in Nigeria — R1/PR6

**DOI:** 10.1017/gmh.2024.25.pr6

**Affiliations:** London School of Hygiene & Tropical Medicine, United Kingdom of Great Britain and Northern Ireland

No accompanying comment.
